# Impressions of the International Dental Congress

**Published:** 1901-04

**Authors:** E. A. Bogue

**Affiliations:** New York


### IMPRESSIONS OF THE INTERNATIONAL DENTAL CONGRESS.[1]

BY DR. E. A. BOGUE, NEW YORK.

A COUNTRY deacon was once called to report upon a site for a graveyard, and he reported that the place selected was so mean that he would never allow himself to be buried there if he lived until he died. You see, this man was called upon to make a report after the work was all done. That is my fix. I promised the chairman to tell a story, and I am now telling one, but I fear I shall have to begin in the middle and leave off at both ends.

And now, to come to the Dental Congress. The management of the preliminaries was most admirable. It may not be known to any of the gentlemen here present that there is one source of ambition to every living Frenchman, and that is to obtain the ribbon of the Legion of Honor. He crawls for it as soon as he gets out of his cradle, and he keeps after it until he gets it or dies a disappointed man. M. Godon was the President of the International Dental Congress, and he, with his secretary, M. Sauvez, worked unceasingly and with success. They so far thought out all the contingencies that might arise that it would be difficult to find anything that they had not thought of and provided for so far as their facilities allowed. Needless to say, M. Godon got his Legion of Honor, and the last time I saw him he had it set in diamonds blazing on the lapel of his coat.

The first meeting of the Congress was held in the building on the exposition grounds devoted to congresses, and of which there were a great many all the way along. There were about twelve hundred names enrolled on the list of membership, and at the first meeting there were probably gathered eight or nine hundred of this number. The subsequent meetings were held in the Hotel of the Scientific Societies, where all the ten sections could meet at the same time and in separate rooms while a general meeting with stereopticon views could be simultaneously run on the ground-floor. The official language, of course, was French, but papers were also read in Italian, German, and English. Difficulties were encoun-

---

[1] Read before The New York Institute of Stomatology, January 4, 1901.

tered by many of the gentlemen present in struggling with these various languages.

Every evening, and generally at noon, entertainments were given either by the committee having the Congress in charge, by the visiting societies or local societies, or by individuals. These entertainments consisted of concerts in the evening, stereopticon exhibitions, dances, and dinners. I had the good fortune to receive an invitation to lunch with about fifty English *confrères,* who received their American brother most cordially. On another occasion Dr. Brophy, Dr. Kirk, Dr. Harlan, and myself were invited to meet a number of English visitors at a dinner at the house of Dr. Burt, himself an Englishman, practising in Paris, whom many of you will remember having been present twelve years ago at the Odontological dinner at the Brunswick. Dr. Burt's dinner was followed by a concert, recitations, etc., and the whole affair was most charming, and illustrative of the kindly feeling that existed regardless of national lines.

A few members of the Dental Congress were invited to a reception tendered to the members of the Medical Congress by President Loubet at the Elysée. I may perhaps be pardoned if I give you a little description of that reception. It does not differ very much, I suppose, from other republican functions, and as none of the gentlemen here present will be able to contradict me, I suppose I can tell such a story as I please. The Elysée palace is the official residence of the French president, as it also was of the last French king. It is a low building with high rooms and very large ones; with several flights of stairs and very big ones. We were ushered past the military guard at the gate, across a court-yard, and half-way up a flight of stairs to a landing, where our invitation cards were taken and we were turned right and left into coat-rooms to dispense with our superfluous belongings, receiving checks instead; then up the rest of the flight of stairs from the landing to the top, where our names were asked or our cards taken and we were introduced into the reception-room, where stood the president and his wife to receive their visitors. I may as well say right here that the Shah of Persia and myself were both moved to pay our respects to the President of France on the same day. The president's officers and attendants, both ladies and gentlemen, were arranged in a sort of semicircle fifteen or twenty feet behind the president and his wife. None of the invited guests remained in this room, but passed on, most of them into the garden and a few of them into other reception-rooms in the building especially arranged for receiving large numbers of people. The garden is about six hundred feet long by two hundred and fifty or three hundred feet wide, surrounded by a brick wall, inside of which is a thick row of trees which completely line the enclosure, and at the corners there is enough shrubbery to form an oval. The centre of this oval was depressed perhaps ten or twelve feet, and from that centre, on every side, extending to the wall was the most beautiful turf, carefully trimmed, and free from all other vegetation; not a weed could I find. The whole centre being clear, a carpet fifteen or twenty feet wide was laid from the palace to the farther end of the garden, where a stage had been erected after the manner of a large theatre, and in front of it there were about two thousand chairs. We had hardly taken our places when the president and his wife and the household, together with the Shah of Persia and his retinue, came down this carpet and took their places in front of this stage. Shortly afterwards the curtain went up on an exhibition of the various dances from the most ancient times up to the latest French craze, under the direction of the ballet-master of the Grand Opera. There were four series of ballets, and the whole thing occupied about two hours and a half. And this is the way in which the French president amused and entertained his guests. After this was over they were still further amused by a slight sprinkling of rain, which caused a skurrying among these people very much as it would happen at a country fair under the same conditions. The rush was in the direction of the palace, where the struggling masses, or such of them as could struggle strongly enough, were served with a collation. I mention this simply to show that all the world is kin, and that the same thing happens at a reception in Paris as would happen at a similar function in New York or Washington. It further interested me to see the large rooms especially provided for receptions, the walls of which were decorated with fine paintings, mural and otherwise, but in which rooms almost no furniture at all had been placed. These rooms would perhaps hold a thousand persons each, and there were several of them.

On Sunday all the members of the Congress were invited to St. Germain for breakfast, and two train-loads went out. We sat down to breakfast at about one o'clock, our usual lunch hour, in a wood attached to a large restaurant and kept for the purpose of providing dinners out of doors and to a large number of people. It was a delightful experience. The members of the Congress and their wives had by this time become more or less acquainted, and the day being so beautiful, they seemed to be in a most perfect condition for enjoying the breakfast under the trees. Tickets were given to each to come back by boat on the Seine, but as the Seine from St. Germain to Paris makes more turns than a double letter S, it takes four hours by boat, so I preferred to come back in another way.

At the Congress it was, of course, impossible to attend more than one of the meetings at a time, although there were ten or eleven going on at once, and hence some of the most valuable contributions escaped me, but the four mentioned on the programme of the evening were those which interested me the most, and it is those which I shall endeavor to briefly describe.

It may be known to you that Dr. Brophy, of Chicago, conceived the idea some twelve or fifteen years ago of operating for cleft palate at the earliest possible stage of infancy. Having done this operation several times, modifications suggested themselves until he reached a point where he earnestly advocated the performance of this operation when the child is but a few days old. It consists in paring the edges of the cleft and putting wire sutures laterally from side to side through the upper maxilla, and then in bending or breaking (at this age fractures probably do not occur) the upper maxilla until the freshened edges are in apposition. The wires on the outside of the gum are passed through pieces of lead and bent over and twisted. The result is that union takes place not only in the soft parts, but in the bony tissue as well, and as time goes on the processes of development seem to throw out these upper maxillæ to almost or quite the dimensions of the normal parts, making it correspond to the lower jaw. Dr. Brophy had several patients at Niagara Falls two or three years ago upon whom he operated when they were infants. I very carefully examined one of them, a girl about twelve years of age, and it was remarkable how nearly the upper jaw corresponded with the lower and how near it was to the normal size. Speech was almost normal; in fact, if one's attention were not called to the fact that it was a case of former cleft palate, it would not have been suspected.

After Dr. Brophy's paper had been read, Professor Gariel, one of the Medical Faculty of Paris, took the floor and acknowledged that, although he was a surgeon, and had operated many times for cleft palate, he had been excessively prejudiced against any method but the one which he had heretofore employed; but that he was quite ready to acknowledge the superiority of Dr. Brophy's method and henceforward to adopt it, especially in view of the facts stated by Dr. Brophy, that out of several hundred cases upon which he had operated, not one had resulted in the death of the patient. Professor Gariel's tribute of respect and admiration to Dr. Brophy was most hearty and very welcome to those who were not only his fellow-countrymen but also fellow-dentists. Professor Gariel's treatment of the whole subject was courteous in the extreme, and his utterances could fairly have been called impassioned, inasmuch as his surgical experience enabled him to accept Dr. Brophy's statements and to adopt his methods. A detailed statement of Dr. Brophy's method of operation will be obtainable by all here present as they read the proceedings of the Congress.

Cognate to this same subject is the method of constructing vela for adult and adolescent cleft palate cases where no operation has been performed. This was brought forward by Mr. E. Lloyd Williams, of London. A model and the apparatus for its construction I have brought with me, and I hope to have the pleasure of demonstrating it to this body at one of its afternoon meetings. Unfortunately, the major part of the apparatus is at this moment at the custom-house, so that we shall have to wait a little. Mr. Williams takes an impression of the mouth and teeth with soft gutta-percha, not going into the cleft. This being chilled with cold water, gives a sharper impression than anything else he has found. The model being made, he strikes up a britannia metal plate of the desired thickness, and with soft solder attaches clasps the same as if it were for artificial teeth. A loop of wire of the general shape, size, and direction of the cleft is then soldered to the posterior edge and upper part of the plate, and to this wire is attached a piece of modelling compound, the recipe for which I have, and hope to give to you later. Then the plate with the modelling compound is placed in the mouth and into the cleft, and the patient is directed to swallow several times. This gives an accurate impression of three sides of the cleft, the two lateral edges and the pharynx. The lingual side of this lump of impressionmaterial must be carved into the shape of an ordinary alveolar arch. The upper side must have some of the impression wax added or cut away until the patient is enabled to produce satisfactory speech. The plate and impression, being removed from the mouth, are duplicated in rubber, the palate part being made into the form of a hollow box, and the whole is vulcanized in a vulcanizer that admits of being screwed down after thirty pounds of steam pressure has been reached and when the rubber is as soft as it can possibly be. This results in a complete piece of work at the third sitting.

Dr. Michaels's paper on Saliva I must not undertake to discuss under these circumstances, because, in the first place, I could not do it justice, and, in the second place, you will get a much more accurate conception of what he is trying to do from the admirable *résumé* in the *Dental Cosmos.* Only this I may say: This is the first time, so far as we know, that careful and scientific investigations have been made of the salivary fluids along these special lines, and the results obtained are striking. Two or three pathological conditions were, it seems, positively recognizable from examination of the saliva without even seeing the patient, and it looks as if some light would be shed upon the processes of dental decay through these investigations. Dr. Michaels has again taken up the study of what used to be known as the chemico-vital processes, and while finding diagnostic symptoms on the one hand, it looks as though the question of dental decay on surfaces which are not defective would also receive additional light.

The last subject to which I would call your attention is the system of crowning and bridging devised by Dr. W. S. Davenport, of Paris. He particularly emphasizes the importance of the accurate fitting of all caps carrying teeth or biting surfaces to the teeth or roots to which they are adapted. These caps should be made of platinum.

Secondly, he advocates the surrounding of each cap with wire from 12 to 20 gauge, 20 being the smallest and generally preferable, in order to flow clasp metal over the wire, thus forming a bulbous tuberosity shaped like a tooth and, while not losing its accurate fit, giving great additional strength.

Thirdly, he advocates one point of attachment and one or more points of rest, which it will be seen does away with the usual manner of firmly cementing two or more teeth together.

Fourthly, the procurement by some means of a perfect occlusion between the bridge and the opposing teeth, so that the balance of parts is thereby restored.

Fifthly, he advocates the use of gutta-percha in solution as the cement for attaching these caps or bridges. He believes that a piece firmly cemented to two or more teeth will, in the course of a few months, break away from all but one of its attachments. The modes of procedure, with illustrations of the process, have been promised to me for the Institute, and I hope to have the pleasure of presenting them at some future period. I only mention it at this time that Dr. Davenport may derive the credit he so richly deserves.

